# Numerical thermal study of ternary nanofluid influenced by thermal radiation towards convectively heated sinusoidal cylinder

**DOI:** 10.1016/j.heliyon.2023.e20057

**Published:** 2023-09-12

**Authors:** Kamel Smida, Adnan, Muhammad Umer Sohail, Iskander Tlili, Asma Javed

**Affiliations:** a Department of General Science and English Language, College of Applied Sciences, AlMaarefa University, Diriyah, 13713, Riyadh, Saudi Arabia; b Departmment of Mathematics, Mohi-ud-Din Islamic University, Nerian Sharif, AJ&K, 12080, Pakistan; c Department of Aeronautics & Astronautics Engineering, Institute of Space Technology, Islamabad, 46000, Pakistan; d Department of Physics, College of Science Al-Zulfi, Majmaah University, Al-Majmaah, 11952, Saudi Arabia; e Department of Computer Science, University of Huddersfield, UK

**Keywords:** Ternary nanofluid, Thermal radiation, Sinusoidal cylinder, Treatment, Nanomaterial

## Abstract

**Applications:**

The heat transfer remains a huge problem for industrialists and engineers because many production processes required considerable amount of heat to finish the process successfully. Although, conventional fluids have large scale industrial applications but unable to provide huge amount of heat transfer. Therefore, the study is organized to propose a new ternary heat transfer model using different physical constraints. The key applications area of nanofluid heat transfer are chemical, applied thermal and food processing engineering.

**Purpose:**

and Methodology: The key purpose of this research is introduce a new ternary nanofluid model using the impressive effects of thermal radiations, surface convection and saddle/nodal points. The results simulated via RKF-45 and discussed in detail.

**Core findings:**

The strength of Al_2_O_3_ nanoparticles form 1%–7% (keeping fixed CuO and Cu as 4% and 6%) and s_1_ = −0.2,-0.4,-0.6,-0.8 controlled the fluid movement while s_1_ = 0.2,0.4,0.6,0.8 boosted the velocity. Increasing the convection process B_i_ = 0.1,0.2,0.3,0.4 increased the temperature significantly. Further, shear drag is maximum for ternary nanofluid and thermal radiations R_d_ = 0.1,0.2,0.3,0.4 enhances the heat transfer rate.

## 1 Introduction

Heat transport is essential many in industrial and engineering disciplines to acquire the desired products. However, working fluids are the key ingredients for this purpose. The conventional fluids are inherently poor thermal performance due to weak thermal conductivity which fail to produce considerable heat for industrial purposes. Therefore, the new fluids introduced with outstanding thermal conductivity and termed as Nanofluids. These fluids are extension of simple fluids with addition of nanoparticles which dispersed in the host functional fluid uniformly and provide significant contribution in thermal transport. Based on the dispersion of nanoparticles, these further characterize as mono nano [1,2], hybrid [3], ternary [4] and tetra nanofluids.

Many industrial apparatuses can be configured by different geometries in which cylinder with plan or wavy surface is significant. In recent decades, researchers and engineers inspired by the applications of heat transfer for sinusoidal and plan cylinders and performed different studies. Recently, Waqas et al. [5] offered numerical computation and simulation of nanofluid transport for cylinder having pores on the surface. The model designed including the effects of solar thermal radiation factor and nanoparticles concentration. Further, higher heat transfer subject to stronger thermal Biot factor was the key conclusion of the study. In 2021, Hussain and Malik [6] conducted in depth analysis of nanofluid comprising microorganism. The working geometry considered as elastic cylinder and MHD, convective and nields conditions included in the biomathematical model. The nanofluids comprising oxide nanoparticles have their own significance due to unique thermal and physical characteristics of nanoparticles. In this regard, a study presented by Rana et al. [7]. They used two-phase approach for nanofluid preparation with slip and MHD effects. The authors introduced the idea of nanolayer and particles diameter using Buongiorno model and examined their impact on the heat transfer ability of the resultant nanofluid.

In 2022, Khan et al. [8] extended the heat transport analysis for hybrid nanoliquids using two sort of nanoparticles. the major physical aspects of the model are the integrated effects of radiative thermal flux and non-uniform internal sink/source. Increment in the drag force and the velocity reduction under increasing internal source factor were the core results of their study. Kabeir et al. [9] encouraged by the transient effects on the heat transfer of nanofluid and endorsed the concept for nanofluid over a cylinder with contracting surface. The transient effects examined through graphical means and provided a comprehensive discussion for the thermal and mass transport mechanism. Some most recent analysis of nanofluid applications from experimental and theoretical aspects presented by different researchers (see Refs. [[10], [11], [12]]). The newtonian heating is an interesting physical insight in the heat transfer modeling and greatly influenced the performance of functional fluid. Thus, this idea convinced Khashiie et al. [13] to perform hybrid nanoliquids analysis for cylinder with resistive heating effects. Mainly the study focused on the heating transport of hybrid nanofluid comprising Cu and Al_2_O_3_ nanoparticles while functional base solvent taken as water.

The heat and mass transportation under magnetic dipole influence for hybrid Casson nanoliquid over an elastic cylinder is accomplished by Ahmad et al. [14]. The Ag and MgO nanoparticles with Casson nanoliquid combined to get the hybrid model and then numerical treatment performed. They examined rapid movement of the fluid particle with enlarging curvature number and opponent behaviour observed for slip and magnetic forces effects. Ramzan et al. [15] investigated the heating ability of Carreau-Yasuda nanofluid with chemical species and upthrust. The achieved model successfully solved via numerical approach and a detailed discussion provided with ranges of parameters. Another analysis for stretchable cylinder with hybrid nanomaterial is inspected by Ali et al. [16] and CuO and ferrite nanoparticle in water mixing was tested for thermal enhancement and analyzed the results graphically. Recently, Waini et al. [17] theoretically reported the characteristics of hybrid nanoliquid over a stretching/shrinking cylinder and concluded that fluid containing two sort of nanoparticles is excellent for thermal enhancement.

Inspired by the enhanced heat transfer rate and cooling performance of nanoliquids, the researchers put their efforts on the study of multiple type of nanoliquids. Recently, Gangadhar et al. [[18], [19], [20], [21]], Marulasiddeshi et al. [22], Wanatasanappan et al. [23], Kotha et al. [24], Bhargavi et al. [25], Kanti et al. [[26], [27], [28]] investigated the heat transfer efficacy of multiple nanoliquids using various geometries effects and the model parameters. The authors focused on both single as well as two phase nanoliquid models. The studies revealed that nanoliquids are more efficient for thermal applications and the results will advantageous to provide sufficient heat to acquire the desired industrial products. In 2019, Hassan et al. [29], Alolaiyan et al. [30], Zeeshan et al. [31,32] and Majeed et al. [33] discussed the dynamics of nanoliquids due to increasing the strength of activation energy, quadratic radiations, Newtonian heating, oxide nanoparticles, magnetic field and internal heating source. Inspection of these studies, it is observed that nanoliquids are good for engineering applications to acquire the target of enhanced heat transfer. Similarly, some of the studies related to nanoliquids that cover the heat transfer applications area are reported by many researchers (see Refs. [[34], [35], [36]]).

According to afore cited literature, it is examined that the previous attempted has been made only up to Newtonian, mono nano and hybrid nanofluids. Thus, there is a significant gap of heat transfer study using novel ternary nanofluid over a sinusoidal cylinder by considering novel ternary nanoliquid as functional fluid over saddle and nodal stagnation region subject to novel effects of convective thermal condition. Three distinct metals nanoparticles namely Al_2_O_3_, CuO and Cu used for the analysis. The heat transfer in ternary nanofluid will be discussed in this specific research under the variable ranges of the parameters involved. The model results would be advantageous for practical applications particularly in applied thermal, chemical and food engineering where enhanced heat transfer is essential. The core focus of this research will.

• To introduce an advance and efficient ternary nanofluid model for sinusoidal cylinder with saddle and nodal stagnation regions.
• To examine the impact of thermal radiation and convective thermal effects on the heat transport efficacy of the functional fluid.
• To examine the influence of particles concentration factor $\varphi_{Al_{2}O_{3},p1}=\varphi_{1},\varphi_{CuO,p2}=\varphi_{2}$ and $\varphi_{Cu,p3}=\varphi_{3}$ on the heat transfer with suitable % ranges.
• To analyze the comparative Skin friction and Nusselt number computation with increasing thermal radiation, Biot number and $\varphi_{i}$ for i=1,2,3.

## 2 Model development

The non-transient 3D laminar stream of ternary nanofluid is considered over a particular cylinder type with sinusoidal radius. Three distinct sorts of nanoparticles and host solvent are the components of functional nanofluid. Due to sinusoidal radius, there exist nodal and saddle points on the cylinder's surface subject to the utmost and minimum radius locations. Further, the coordinate directions [x, y, z] associated with the velocity components [u, v, w]. Further, ${\underline{u}}_{e}=\alpha_{1}\underline{x}$ and ${\underline{v}}_{e}=\alpha_{2}\underline{y}$ in which $\alpha_{1}$ and $\alpha_{2}$ are the constants connected with free stream. The equation ${\underline{x}}_{e}=\alpha_{3}^{1/\underline{c}}$ where $\underline{c}$ is the quotient of $\alpha_{2}$ to $\alpha_{1}$, and constant $\alpha_{3}$ offers specific streamlines. The ranges of stagnation point for saddle and nodal points are $-1.0<\underline{c}<0.0$ and $1.0<\underline{c}<1.0$, respectively. If $\underline{c}=0$ then the saddle and nodal locations approach to the cylinder surface with constant radius.

### 2.1 Necessary assumptions

The model under consideration will be associated to the subsequent assumptions.

• The flow is laminar, non-transient and 3D over a cylinder with non-uniform radius.
• The surface is considered with no thermal and velocity slip.
• The flow is SPF and the streamlines separated through saddle and nodal points.
• No chemical reaction takes place and the nanoparticles (Al_2_O_3_–CuO–Cu) uniformly saturated in the host solvent.

### 2.2 Model geometry

**Fig. 1:**
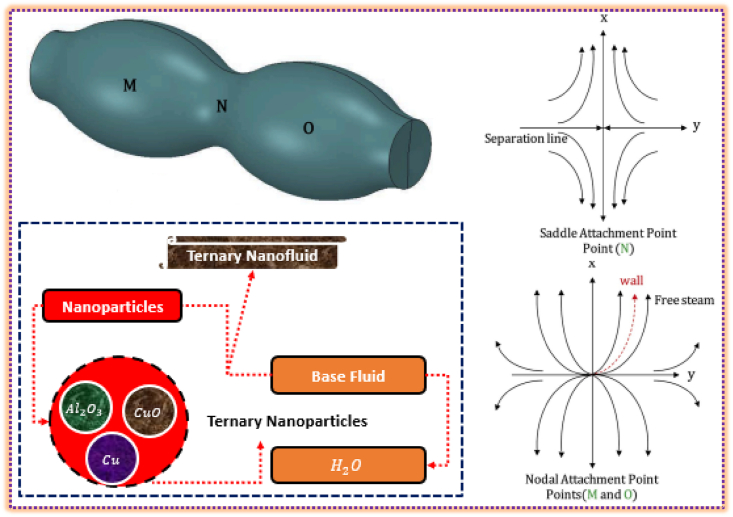
The ternary nanofluid flow through a cylinder with variable radius.

The above SPF of ternary nanofluid past a sinusoidal cylinder physically described in Fig. 1 with saddle and nodal points location. The figure also describes the separation line and freestream for saddle and nodal attachments.

### 2.3 Governing laws associated to the model

The primary governing laws for 3D laminar flow of ternary nanofluid over a sinusoidal radius describe by the following physical rules [37,38]:

(1) $${\underline{u}}_{x}+{\underline{v}}_{y}+{\underline{w}}_{z}=0$$

(2) $$\underline{u}\;{\underline{u}}_{x}+\underline{v}\;{\underline{u}}_{y}+\underline{w}\;{\underline{u}}_{z}=\alpha_{1}x+\frac{\mu_{ternary}}{\rho_{ternary}}\left[{\underline{u}}_{zz}\right]$$

(3) $$\underline{u}\;{\underline{v}}_{x}+\underline{v}\;{\underline{v}}_{y}+\underline{w}\;{\underline{v}}_{z}=\alpha_{2}y+\frac{\mu_{ternary}}{\rho_{ternary}}\left[{\underline{v}}_{zz}\right]$$

(4) $$\underline{u}\;{\underline{T}}_{x}+\underline{v}\;{\underline{T}}_{y}+\underline{w}\;{\underline{T}}_{z}=\frac{k_{ternary}}{{\left(\rho c_{p}\right)}_{ternary}}\left[{\underline{T}}_{zz}\right]+\frac{16\sigma^{*}{\underline{T}}_{\infty }^{3}}{3k^{*}{\left(\rho c_{p}\right)}_{ternary}}\left[{\underline{T}}_{zz}\right]$$

Further, the specified boundary and ambient location of the cylinder are as follows [38]:

(5) $$\underline{u}=0,\underline{v}=0,\underline{w}=0,k_{ternary}{\underline{T}}_{z}=h_{f}\left({\underline{T}}_{f}-\underline{T}\right)\;when\;z=0$$

(6) $$\underline{u}\to {\underline{u}}_{e},\underline{v}\to {\underline{v}}_{e},\underline{T}\to {\underline{T}}_{\infty }\;when\;z\to \infty$$

Further, in order to transform the model in more simplified form, the following appropriate similarities for sinusoidal cylinder will be adopted:

(7) $$\underline{u}=\alpha_{1}xF^{\prime },\underline{v}=\alpha_{2}yG^{\prime },w=\sqrt{\alpha_{1}\nu_{f}}\;\left[F+cG\right],\underline{T}={\underline{T}}_{\infty }+\left[{\underline{T}}_{w}-{\underline{T}}_{\infty }\right]\beta ,\eta =z\sqrt{\frac{\nu_{f}}{a}}$$

Here, $c_{1}=\frac{\alpha_{2}}{\alpha_{1}}$ and the functions F,G and β depend on the similarity variable η.

### 2.4 Supporting nanofluid models and thermophysical characteristics

The traditional model of newtonian fluid over a sinusoidal cylinder will be modified using the below advance ternary nanofluid [39] (Table 1) and thermophysical characteristics. It is noteworthy that the previous model (hybrid, nano and common fluids) can be achieved by setting concentration factor.

**Table 1:** Thermal and Physical characteristics of ternary nanofluid, nanoparticles and host solvent.

Property	Expression	Supporting Empirical Expression			
Heat Capacity	$\frac{{\left(\rho c_{p}\right)}_{ternary}}{{\left(\rho c_{p}\right)}_{f}}$	$\left(1-\varphi_{Cu,p3}\right)\left[\left(1-\varphi_{CuO,p2}\right)\left[\left(1-\varphi_{Al_{2}O_{3},p1}\right)+\varphi_{Al_{2}O_{3},p1}\frac{{\left(\rho c_{p}\right)}_{Al_{2}O_{3},p1}}{{\left(\rho c_{p}\right)}_{H_{2}O}}\right]+\varphi_{Al_{2}O_{3},p1}\frac{{\left(\rho c_{p}\right)}_{CuO,p2}}{{\left(\rho c_{p}\right)}_{H_{2}O}}\right]+\varphi_{Cu,p3}\frac{{\left(\rho c_{p}\right)}_{Cu,p3}}{{\left(\rho c_{p}\right)}_{H_{2}O}}$			
Density	$\frac{\rho_{ternary}}{\rho_{f}}$	$\left(1-\varphi_{Cu,p3}\right)\left[\left(1-\varphi_{CuO,p2}\right)\left[\left(1-\varphi_{Al_{2}O_{3},p1}\right)+\varphi_{Al_{2}O_{3},p1}\frac{{\left(\rho \right)}_{Al_{2}O_{3},p1}}{{\left(\rho \right)}_{H_{2}O}}\right]+\varphi_{Al_{2}O_{3},p1}\frac{{\left(\rho \right)}_{CuO,p2}}{{\left(\rho \right)}_{H_{2}O}}\right]+\varphi_{Cu,p3}\frac{{\left(\rho \right)}_{Cu,p3}}{{\left(\rho \right)}_{H_{2}O}}$			
Dynamic Viscosity	$\frac{\mu_{ternary}}{\mu_{f}}$	$\frac{1}{{\left[1-\varphi_{Al_{2}O_{3},p1}\right]}^{2.5}{\left[1-\varphi_{CuO,p2}\right]}^{2.5}{\left[1-\varphi_{Cu,p3}\right]}^{2.5}}$			
Thermal Conductivity	$\frac{k_{ternary}}{k_{f}}$	$\frac{{\overset{˘}{k}}_{\left(Al_{2}O_{3}-CuO-Cu\right)w}}{{\overset{˘}{k}}_{\left(Al_{2}O_{3}-CuO\right)w}}=\frac{{\overset{˘}{k}}_{Cu,p3}+2{\overset{˘}{k}}_{\left(Al_{2}O_{3}-CuO\right)w}-2\varphi_{Cu,p3}\left({\overset{˘}{k}}_{\left(Al_{2}O_{3}-CuO\right)w}-{\overset{˘}{k}}_{Cu,p3}\right)}{{\overset{˘}{k}}_{Cu,p3}+{2\overset{˘}{k}}_{\left(Al_{2}O_{3}-CuO\right)w}+\varphi_{Cu,p3}\left({\overset{˘}{k}}_{\left(Al_{2}O_{3}-CuO\right)w}-{\overset{˘}{k}}_{Cu,p3}\right)}$ $\frac{k_{\left(Al_{2}O_{3}-CuO\right)w}}{k_{nf}}=\frac{{\overset{˘}{k}}_{CuO,p2}+2{\overset{˘}{k}}_{nf}-2\varphi_{CuO,p2}\left({\overset{˘}{k}}_{nf}-{\overset{˘}{k}}_{CuO,p2}\right)}{{\overset{˘}{k}}_{CuO,p2}+2{\overset{˘}{k}}_{nf}+\varphi_{CuO,p2}\left({\overset{˘}{k}}_{nf}-{\overset{˘}{k}}_{CuO,p2}\right)}$ $\frac{k_{nf}}{k_{f}}=\frac{{\overset{˘}{k}}_{Al_{2}O_{3},p1}+2{\overset{˘}{k}}_{H_{2}O}-2\varphi_{Al_{2}O_{3,p1}}\left({\overset{˘}{k}}_{H_{2}O}-{\overset{˘}{k}}_{Al_{2}O_{3},p1}\right)}{{\overset{˘}{k}}_{Al_{2}O_{3,p1}}+2{\overset{˘}{k}}_{H_{2}O}+\varphi_{Al_{2}O_{3,p1}}\left({\overset{˘}{k}}_{H_{2}O}-{\overset{˘}{k}}_{Al_{2}O_{3,p1}}\right)}$			
Electrical Conductivity	$\frac{\sigma_{ternary}}{\sigma_{f}}$	$\frac{{\overset{˘}{\sigma }}_{\left(Al_{2}O_{3}-CuO-Cu\right)H_{2}O}}{{\overset{˘}{\sigma }}_{\left(Al_{2}O_{3}-CuO\right)H_{2}O}}=\frac{{\overset{˘}{\sigma }}_{Cu,p3}+2{\overset{˘}{\sigma }}_{\left(Al_{2}O_{3}-CuO\right)w}-2\varphi_{Cu,p3}\left({\overset{˘}{\sigma }}_{\left(Al_{2}O_{3}-CuO\right)w}-{\overset{˘}{\sigma }}_{Cu,p3}\right)}{{\overset{˘}{\sigma }}_{Cu,p3}+2{\overset{˘}{\sigma }}_{\left(Al_{2}O_{3}-CuO\right)w}+\varphi_{Cu,p3}\left({\overset{˘}{\sigma }}_{\left(Al_{2}O_{3}-CuO\right)w}-{\overset{˘}{\sigma }}_{Cu,p3}\right)}$ where $\frac{{\overset{˘}{\sigma }}_{\left(Al_{2}O_{3}-CuO\right)w}}{{\overset{˘}{\sigma }}_{nf}}=\frac{{\overset{˘}{\sigma }}_{CuO,p2}+2{\overset{˘}{\sigma }}_{nf}-2\varphi_{CuO,p2}\left({\overset{˘}{\sigma }}_{nf}-{\overset{˘}{\sigma }}_{CuO,p2}\right)}{{\overset{˘}{\sigma }}_{CuO,p2}+2{\overset{˘}{\sigma }}_{nf}+\varphi_{CuO,p2}\left({\overset{˘}{\sigma }}_{nf}-{\overset{˘}{\sigma }}_{CuO,p2}\right)}$ $\frac{{\overset{˘}{\sigma }}_{nf}}{{\overset{˘}{\sigma }}_{H_{2}O}}=\frac{{\overset{˘}{\sigma }}_{Al_{2}O_{3}},p1+2{\overset{˘}{\sigma }}_{H_{2}O}-2\varphi_{Al_{2}O_{3},p1}\left({\overset{˘}{\sigma }}_{H_{2}O}-{\overset{˘}{\sigma }}_{Al_{2}O_{3}},p1\right)}{{\overset{˘}{\sigma }}_{Al_{2}O_{3},p1}+2{\overset{˘}{\sigma }}_{H_{2}O}+\varphi_{Al_{2}O_{3},p1}\left({\overset{˘}{\sigma }}_{H_{2}O}-{\overset{˘}{\sigma }}_{Al_{2}O_{3},p1}\right)}$			
Basic Components		$\hat{\rho }\left(kg/m^{3}\right)$	${\hat{c}}_{p}\left(J/Kg\;K\right)$	$\hat{k}\left(W/mk\right)$	$\overset{˘}{\sigma }{\left(\Omega m\right)}^{-1}$
CuO		6500	540	18	$6.9\times 10^{-2}$
H_2_O		997.1	4180	0.6071	$5.5\times 10^{-6}$
Cu		8933	385	400	$59.6\times 10^{6}$
Al_2_O_3_		3970	765	40	$35\times 10^{6}$

### 2.5 Final heat transfer model for sinusoidal cylinder

This subsection deals with the final version of the enhanced heat transfer over a sinusoidal cylinder. For this, we used the appropriate model similarity variables and supporting ternary nanofluid expressions in the basic governing laws mentioned in Eqs. (1), (2), (3), (4). Thus, the final form of the model given in Eqs. (8), (9), (10).

(8) $$\frac{{\left[\left(1-\varphi_{Cu,p3}\right)\left[\left(1-\varphi_{CuO,p2}\right)\left[\left(1-\varphi_{Al_{2}O_{3},p1}\right)+\varphi_{Al_{2}O_{3},p1}\frac{{\left(\rho \right)}_{Al_{2}O_{3},p1}}{{\left(\rho \right)}_{H_{2}O}}\right]+\varphi_{Al_{2}O_{3},p1}\frac{{\left(\rho \right)}_{CuO,p2}}{{\left(\rho \right)}_{H_{2}O}}\right]+\varphi_{Cu,p3}\frac{{\left(\rho \right)}_{Cu,p3}}{{\left(\rho \right)}_{H_{2}O}}\right]}^{-1}}{{\left[1-\varphi_{Al_{2}O_{3},p1}\right]}^{2.5}{\left[1-\varphi_{CuO,p2}\right]}^{2.5}{\left[1-\varphi_{Cu,p3}\right]}^{2.5}}F^{\prime \prime \prime }+\left[s_{1}F^{\prime \prime }G-F^{\prime 2}+1+F^{\prime \prime }F\right]=0$$

(9) $$\frac{{\left[\left(1-\varphi_{Cu,p3}\right)\left[\left(1-\varphi_{CuO,p2}\right)\left[\left(1-\varphi_{Al_{2}O_{3},p1}\right)+\varphi_{Al_{2}O_{3},p1}\frac{{\left(\rho \right)}_{Al_{2}O_{3},p1}}{{\left(\rho \right)}_{H_{2}O}}\right]+\varphi_{Al_{2}O_{3},p1}\frac{{\left(\rho \right)}_{CuO,p2}}{{\left(\rho \right)}_{H_{2}O}}\right]+\varphi_{Cu,p3}\frac{{\left(\rho \right)}_{Cu,p3}}{{\left(\rho \right)}_{H_{2}O}}\right]}^{-1}}{{\left[1-\varphi_{Al_{2}O_{3},p1}\right]}^{2.5}{\left[1-\varphi_{CuO,p2}\right]}^{2.5}{\left[1-\varphi_{Cu,p3}\right]}^{2.5}}G^{\prime \prime \prime }+\left[G^{\prime \prime }F+s_{1}G^{\prime \prime }G-s_{1}G^{\prime 2}+s_{1}\right]=0$$

(10) $$\left(1+\frac{R_{d}}{\frac{k_{ternary}}{k_{f}}}\right)\beta^{\prime \prime }+\frac{\frac{{\left(\rho c_{p}\right)}_{ternary}}{{\left(\rho c_{p}\right)}_{f}}}{\frac{k_{ternary}}{k_{f}}}\mathrm{Pr}\left(F\beta^{\prime }+c_{1}\beta^{\prime }G\right)=0$$

where;

$$\frac{{\left(\rho c_{p}\right)}_{ternary}}{{\left(\rho c_{p}\right)}_{f}}=\left(1-\varphi_{Cu,p3}\right)\left[\left(1-\varphi_{CuO,p2}\right)\left[\left(1-\varphi_{Al_{2}O_{3},p1}\right)+\varphi_{Al_{2}O_{3},p1}\frac{{\left(\rho c_{p}\right)}_{Al_{2}O_{3},p1}}{{\left(\rho c_{p}\right)}_{H_{2}O}}\right]+\varphi_{Al_{2}O_{3},p1}\frac{{\left(\rho c_{p}\right)}_{CuO,p2}}{{\left(\rho c_{p}\right)}_{H_{2}O}}\right]+\varphi_{Cu,p3}\frac{{\left(\rho c_{p}\right)}_{Cu,p3}}{{\left(\rho c_{p}\right)}_{H_{2}O}}$$

$$\frac{k_{ternary}}{k_{f}}=\left[\begin{array}{c} \frac{{\overset{˘}{k}}_{Cu,p3}+2{\overset{˘}{k}}_{\left(Al_{2}O_{3}-CuO\right)w}-2\varphi_{Cu,p3}\left({\overset{˘}{k}}_{\left(Al_{2}O_{3}-CuO\right)w}-{\overset{˘}{k}}_{Cu,p3}\right)}{{\overset{˘}{k}}_{Cu,p3}+{2\overset{˘}{k}}_{\left(Al_{2}O_{3}-CuO\right)w}+\varphi_{Cu,p3}\left({\overset{˘}{k}}_{\left(Al_{2}O_{3}-CuO\right)w}-{\overset{˘}{k}}_{Cu,p3}\right)}*\\ \frac{{\overset{˘}{k}}_{CuO,p2}+2{\overset{˘}{k}}_{nf}-2\varphi_{CuO,p2}\left({\overset{˘}{k}}_{nf}-{\overset{˘}{k}}_{CuO,p2}\right)}{{\overset{˘}{k}}_{CuO,p2}+2{\overset{˘}{k}}_{nf}+\varphi_{CuO,p2}\left({\overset{˘}{k}}_{nf}-{\overset{˘}{k}}_{CuO,p2}\right)}*\\ \frac{{\overset{˘}{k}}_{{Al}_{2}O_{3},p1}+2{\overset{˘}{k}}_{f}-2\varphi_{{Al}_{2}O_{3},p1}\left({\overset{˘}{k}}_{f}-{\overset{˘}{k}}_{{Al}_{2}O_{3},p1}\right)}{{\overset{˘}{k}}_{{Al}_{2}O_{3},p1}+2{\overset{˘}{k}}_{f}+\varphi_{{Al}_{2}O_{3},p1}\left({\overset{˘}{k}}_{f}-{\overset{˘}{k}}_{{Al}_{2}O_{3},p1}\right)} \end{array}\right]$$

In Eqs. (8), (9), $\varphi_{Al_{2}O_{3},p1}=\varphi_{1},\varphi_{CuO,p2}=\varphi_{2}$ and $\varphi_{Cu,p3}=\varphi_{3}$ which are concentration factor of the nanoparticles. Now, the dimensionless version of transformed BC's is as follows:

(11) $$F\left(\eta \right)=0,F^{\prime }\left(\eta \right)=0,G\left(\eta \right)=0,G^{\prime }\left(\eta \right)=0,\beta^{\prime }\left(\eta \right)=-B_{i}\left[\beta \left(\eta \right)-1\right]\;at\;\eta =0$$

(12) $$F^{\prime }\left(\eta \right)\to 1,G^{\prime }\left(\eta \right)\to 1,\beta \left(\eta \right)\to 0\;when\;\eta \to \infty$$

Further, physical quantities integrated in the model are Prandtl number ($\mathrm{Pr}=\nu_{f}\alpha_{f}^{-1}$), thermal radiation ($R_{d}=\frac{16\sigma^{*}{\underline{T}}_{\infty }^{3}}{3kk^{*}}$) and Biot number $B_{i}$.

### 2.6 Quantities of interest

Important physical quantities for engineering interest are Nusselt number and Skin friction. These quantities significantly change due physical factor described in the model and appropriate formulae are as under:

(13) $$C_{Fx}=\tau_{wz}\left[\rho_{f}{\underline{u}}_{w}^{2}\right],C_{Fy}=\tau_{wy}\left[\rho_{f}{\underline{u}}_{w}^{2}\right],and\;N_{ux}=\frac{x}{q_{w}^{-1}k_{f}\left[{\underline{T}}_{w}-{\underline{T}}_{\infty }\right]}$$

Now, the supporting shear stresses and $q_{w}$ defined in the formulae given in Eq. (14).

(14) $$\tau_{wx}=\mu_{ternary}{\left[{\underline{u}}_{z}\right]}_{z=0},\tau_{wy}=\mu_{ternary}{\left[{\underline{v}}_{y}\right]}_{z=0},q_{w}=-k_{ternary}{\left[{\underline{T}}_{z}\right]}_{z=0}$$

with the help of Eqs. (13), (14), the following resultant expressions obtained after performing mathematical operations:

(15) $${\left[R_{ex}\right]}^{1/2}C_{Fx}=\mu_{\frac{ternary}{\mu_{f}}}F^{\prime \prime }\left(0\right),{\left[R_{ex}\right]}^{1/2}C_{Fy}=\mu_{\frac{ternary}{\mu_{f}}}{c_{1}G}^{\prime \prime }\left(0\right)$$

(16) $$N_{ux}{\left[R_{ex}\right]}^{-1.2}=-\frac{k_{ternary}}{k_{f}}\beta^{\prime }\left(0\right)$$

In the above Eqs. (15), (16), the expression $\frac{\mu_{ternary}}{\mu_{f}}$ and $\frac{k_{ternary}}{k_{f}}$ described in Table 1.

## 3 Mathematical investigation of the model

**Fig. 2:**
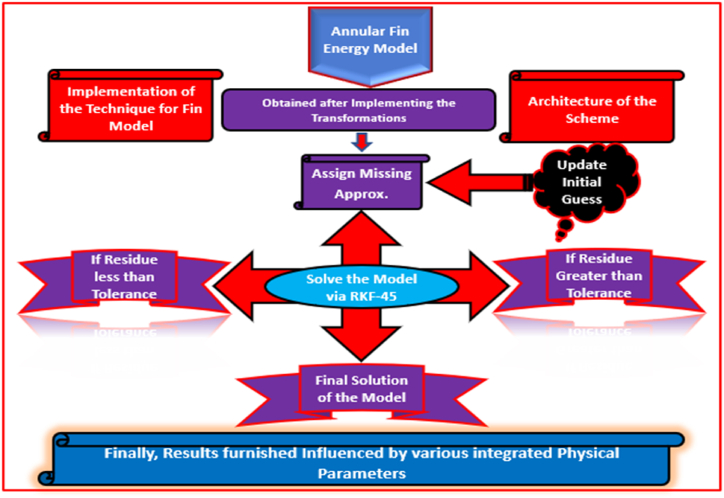
The mathematical procedure of RKF-45 for ternary model over a sinusoidal cylinder.

Under consideration model comprises high nonlinearities due to which the exact solution is not appropriate. Thus, the RKF-45 numerical scheme (see Refs. [[40], [41], [42], [43], [44], [45]]) adopted for the solution and to investigate the impact of ingrained physical constraints. In this scheme, initial transformations essential to reduce the model into IVP (see Refs. [[46], [47], [48], [49], [50]]) and then proceed with solution of the system. The whole solution procedure under this scheme is elaborated in Fig. 2.

The supporting transformations for the model conversion are as under:

(17) $$\left[{⋌}_{1},{⋌}_{2},{⋌}_{3},{⋌}_{3}^{\prime },{⋌}_{4},{⋌}_{5},{⋌}_{6},{⋌}_{6}^{\prime },{⋌}_{7},{⋌}_{8},{⋌}_{8}^{\prime }\right]=\left[F,F^{\prime },F^{\prime \prime },F^{\prime \prime \prime },G,G^{\prime },G^{\prime \prime },G^{\prime \prime \prime },\beta ,\beta^{\prime },\beta^{\prime \prime }\right]$$

Now, proceed to the proper arrangement of the model and using the above transformations, below system achieved.

(18) $$\left[\begin{array}{c} {⋌}_{3}^{\prime }\\ {⋌}_{6}^{\prime }\\ {⋌}_{8}^{\prime } \end{array}\right]=\left[\begin{array}{c} -\varpi_{1}\left(s_{1}{⋌}_{3}{⋌}_{4}-{⋌}_{2}^{2}+1+{⋌}_{1}{⋌}_{3}\right)\\ -\varpi_{2}\left({⋌}_{6}{⋌}_{1}+s_{1}{⋌}_{6}{⋌}_{4}-s_{1}{⋌}_{5}^{2}+s_{1}\right)\\ -{\left(1+\frac{R_{d}}{\frac{k_{ternary}}{k_{f}}}\right)}^{-1}\left(\frac{\frac{{\left(\rho c_{p}\right)}_{ternary}}{{\left(\rho c_{p}\right)}_{f}}}{\frac{k_{ternary}}{k_{f}}}\mathrm{Pr}\left({⋌}_{1}{⋌}_{8}+s_{1}{⋌}_{4}{⋌}_{8}\right)\right) \end{array}\right]$$

where; $\varpi_{1}$ and $\varpi_{2}$ define the following thermal and physical attributes of the ternary nanofluid.

$$\varpi_{1}=\frac{1}{\frac{{\left[\left(1-\varphi_{Cu,p3}\right)\left[\left(1-\varphi_{CuO,p2}\right)\left[\left(1-\varphi_{Al_{2}O_{3},p1}\right)+\varphi_{Al_{2}O_{3},p1}\frac{{\left(\rho \right)}_{Al_{2}O_{3},p1}}{{\left(\rho \right)}_{H_{2}O}}\right]+\varphi_{Al_{2}O_{3},p1}\frac{{\left(\rho \right)}_{CuO,p2}}{{\left(\rho \right)}_{H_{2}O}}\right]+\varphi_{Cu,p3}\frac{{\left(\rho \right)}_{Cu,p3}}{{\left(\rho \right)}_{H_{2}O}}\right]}^{-1}}{{\left[1-\varphi_{Al_{2}O_{3},p1}\right]}^{2.5}{\left[1-\varphi_{CuO,p2}\right]}^{2.5}{\left[1-\varphi_{Cu,p3}\right]}^{2.5}}}$$

$$\varpi_{2}=\frac{1}{\frac{{\left[\left(1-\varphi_{Cu,p3}\right)\left[\left(1-\varphi_{CuO,p2}\right)\left[\left(1-\varphi_{Al_{2}O_{3},p1}\right)+\varphi_{Al_{2}O_{3},p1}\frac{{\left(\rho \right)}_{Al_{2}O_{3},p1}}{{\left(\rho \right)}_{H_{2}O}}\right]+\varphi_{Al_{2}O_{3},p1}\frac{{\left(\rho \right)}_{CuO,p2}}{{\left(\rho \right)}_{H_{2}O}}\right]+\varphi_{Cu,p3}\frac{{\left(\rho \right)}_{Cu,p3}}{{\left(\rho \right)}_{H_{2}O}}\right]}^{-1}}{{\left[1-\varphi_{Al_{2}O_{3},p1}\right]}^{2.5}{\left[1-\varphi_{CuO,p2}\right]}^{2.5}{\left[1-\varphi_{Cu,p3}\right]}^{2.5}}}$$

The updated model is then analyzed using RK scheme with tolerance up to $10^{-6}$. From, the results point of view accuracy of the scheme is subject to asymptotic behaviour (ambient BCs) of the velocity and thermal profiles under various parametric stages.

## 4 Results and discussion

The effects of the physical constraints like particles strength $\varphi_{Al_{2}O_{3},p1}=\varphi_{1},\varphi_{CuO,p2}=\varphi_{2}$ and $\varphi_{Cu,p3}=\varphi_{3}$ and thermal radiation $R_{d}$ on the fluid movement, temperature, shear drag and heat transfer rate over the wavy surface of cylinder are simulated in this section. Further, tabulated results along with computational cost are tabulated against different parameters values.

### 4.1 The velocity fields $F^{\prime }$ and $G^{\prime }$

**Fig. 3:**
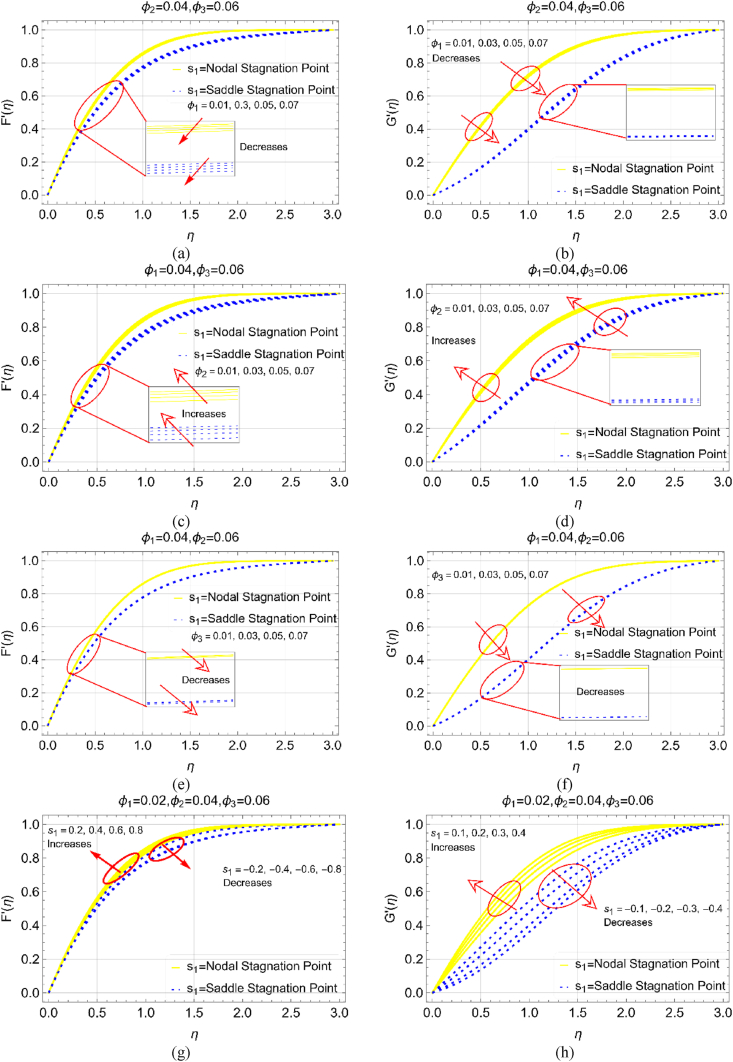
The velocity fields ($F^{\prime }$ and $G^{\prime }$) variations for increasing (a, b) $\varphi_{1}$ (c, d) $\varphi_{2}$ (e, f) $\varphi_{3}$ and (g, h) $s_{1}$ for nodal and saddle points.

The effects of concentration factors ($\varphi_{Al_{2}O_{3},p1}=\varphi_{1},\varphi_{CuO,p2}=\varphi_{2}$ and $\varphi_{Cu,p3}=\varphi_{3}$) and $s_{1}$ on the velocities $F^{\prime }$ and $G^{\prime }$ are demonstrated in Fig. 3a-h. It is examined that the fluid motion reduces by increasing the nanoparticles strength (Fig. 3a and b). Physically, viscous forces and density of the functional fluid rises when more particles added in the common solvent. Due to this reason, the resistive forces opposes the fluid movement over the surface including saddle and nodal points. These variations observed for increasing $\varphi_{Al_{2}O_{3},p1}=\varphi_{1}$ while $\varphi_{CuO,p2}=\varphi_{2}$ and $\varphi_{Cu,p3}=\varphi_{3}$ are fixed at 6%. In Fig. 3c and d the velocity improved as the concentration values of $\varphi_{CuO,p2}=\varphi_{2}$ vary from 1% to 6% while holding $\varphi_{CuO,p2}=\varphi_{2}$ and $\varphi_{Cu,p3}=\varphi_{3}$ as 4%. Increasing the concentration factor of the second nanoparticles in the fluid mixture, the fluid particles move rapidly. Physically, the viscous forces in the adjacent fluid layers become weaker due to which the fluid moves freely over the saddle and nodal locations of the cylinder surface.

The fluid movement for varying $\varphi_{Cu,p3}=\varphi_{3}$ and $s_{1}$ is elaborated in Fig. 3e–f and Fig. 3g–h, respectively. The increment in $\varphi_{Cu,p3}=\varphi_{3}$ fully resists the movement for both $F^{\prime }$ and $G^{\prime }$. Physically, more fluid molecules drag to fill the gap over the surface which ultimately opposes the motion. At free stream position, the velocity approaches to 1 while keeping $\eta_{\max}=3.0$. Similarly, Fig. 3g and h elucidates the movement for variable saddle and nodal stagnation point locations. This time reverse velocity trends examined from both the graphical scenarios. This shows that sinusoidal radius (saddle and nodal stagnation locations) is significant to control and enhance the ternary nanofluid movement past through the cylinder surface. For nodal stagnation point (as mentioned in Fig. 1), the fluid motion is dominant wheraes; saddle region of the cylinder resists the ternary nanoliquid motion. The particles concentration is holding at $\varphi_{Al_{2}O_{3},p1}=\varphi_{1}=2\%,\varphi_{CuO,p2}=\varphi_{2}=4\%$ and $\varphi_{Cu,p3}=\varphi_{3}=6\%$.

### 4.2 Thermal field under varying parameters

**Fig. 4:**
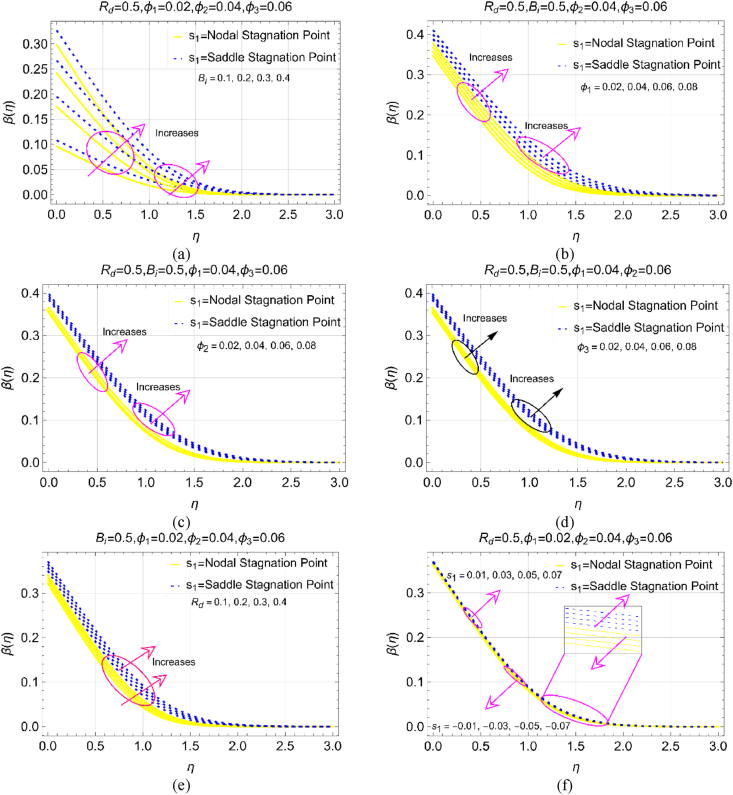
Thermal field for increasing values of (a) $B_{i}$ (b) $\varphi_{1}$ (c) $\varphi_{2}$ (d) $\varphi_{3}$ (e) $R_{d}$ and (f) $s_{1}$.

The nanofluids are of key interest due to enrich thermal conductivity and their role is significant for thermal enhancement. The model physical constraints greatly influence thermal transport in ternary nanoliquids. Therefore, this sub section organized to examine the thermal field trends in ternary nanoliquid over as sinusoidal cylinder. Influence of $B_{i}$ due to convective cylinder surface, particles concentration ($\varphi_{Al_{2}O_{3},p1}=\varphi_{1},\varphi_{CuO,p2}=\varphi_{2}$ and $\varphi_{Cu,p3}=\varphi_{3}$), radiation factor $R_{d}$ and $s_{1}$ are demonstrated in Fig. 4a–f, respectively.

Fig. 4a and b deals with the impact of $B_{i}$ and φ on β(η) ranging from 0.1 to 0.4 and 2%–8%, respectively. The results witness that the temperature of ternary nanoliquid intensifies while $B_{i}$ increases. Physically, higher $B_{i}$ values indicate the stronger convection from the surface. The fast convection process transmits larger amount of heat to the successive ternary fluid layers which leads to significant increment in the fluid temperature. At the cylinder's surface (η=0) over the saddle and nodal stagnation points ($s_{1}<0$ and $s_{1}>0$) the temperature variations are very high as the particles near the surface become more heated under stronger convection effects. Further, the thermal boundary layer region (TBLR) for both saddle and nodal locations observed beyond η=2.0 in which the temperature follows the ambient thermal condition i.e., β→0.0 as η→3.0.

In the analysis of ternary nanoliquids, another important factor is the settlement of particles concentration in the host solvent. This factor is like a catalytic process in the temperature enhancement ($\varphi_{Al_{2}O_{3},p1}=\varphi_{1},\varphi_{CuO,p2}=\varphi_{2}$ and $\varphi_{Cu,p3}=\varphi_{3}$), of nanoliquids. Thus, Fig. 4b–d demonstrates the temperature variations under the particle strength. The high amount of heat transfer is examined for $\varphi_{Al_{2}O_{3},p1}=\varphi_{1}$ against 2%–8% keeping the other two $\varphi_{2}$ and $\varphi_{3}$ fixed in Fig. 4b. Although, the temperature increases with increase in concentration of $\varphi_{CuO,p2}=\varphi_{2}$ and $\varphi_{Cu,p3}=\varphi_{3}$ but these effects are minimal than the concentration of $Al_{2}O_{3}$. Physically, additional thermal conductivity of nanoparticles enhances the heat transmission of resultant nanoliquid thus; the temperature rises significantly.

Addition of thermal radiation effects in the model is a natural and vital source which is important for thermal enhancement applications in nanofluids. These effects under various stages are demonstrated in Fig. 4e and f deals with the impact of $s_{1}$. It is noticed that high thermal radiative number $R_{d}$ increases the ternary nanoliquid temperature. Physically, directed thermal radiations factor endorsed heat to the fluid particles and the neighboring particles gain energy from other particles as a consequence the temperature upsurges. Further, almost insignificant role of $s_{1}$ is examined for thermal behaviour of ternary nanoliquid over a sinusoidal cylinder (Fig. 4f).

**Fig. 5:**
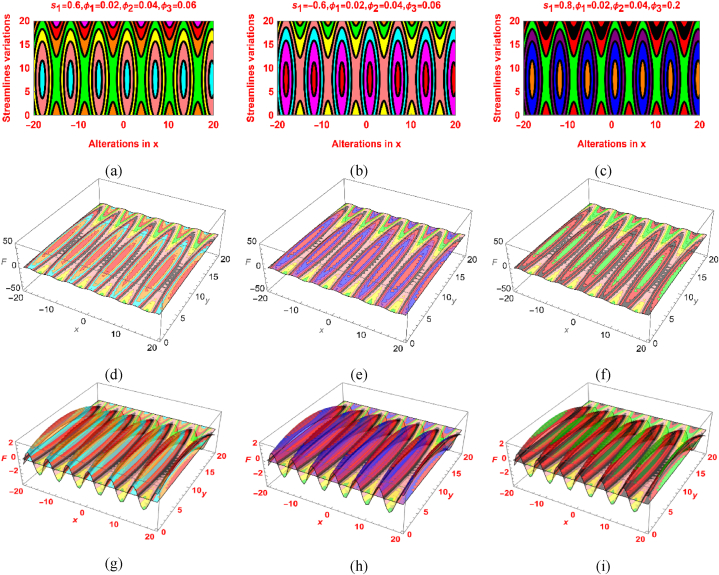
The Streamlines variations for (a) $s_{1}=0.6$ (b) $s_{1}=-0.6$ (c) $s_{1}=0.8$ and 2D scenario in (d, e, f) and 3D scenario in (g, h, i).

**Fig. 6:**
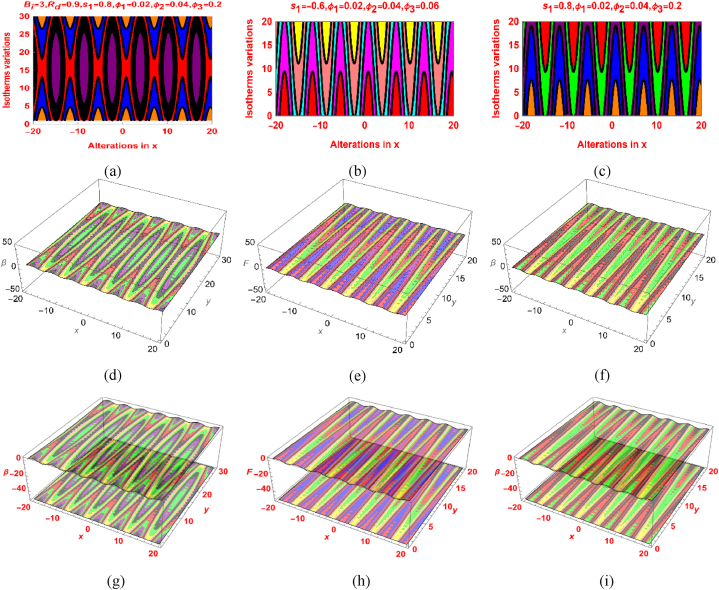
Isotherm variations for (a) $s_{1}=0.6$ (b) $s_{1}=-0.6$ (c) $s_{1}=0.8$ and 2D scenario in (d, e, f) and 3D scenario in (g, h, i).

Fig. 5a–i and Fig. 6a–i are demonstrating the fascinating variations in the streamlines and isotherms under the particles concentration and other physical constraints. These trends plotted for $s_{1}=0.6,s_{1}=-0.6$ and $s_{1}=0.8$ which indicates different nodal and saddle points locations. It can be seen that the isotherms pattern expanded and fluid layers pattern squeeze as the values of the parameters increases. Further, the 2D and 3D pattern is also furnished in Fig. 5, Fig. 6.

### 4.3 Tables discussion

Table 2 and Table 3 contain a detailed computation for skin friction $\sqrt{R_{ex}}\;C_{Fx}$ and $\sqrt{R_{ex}}\;C_{Fy}$ with increasing particles concentration. The computational cost against the computed values is also provided at different stages. Table 2 reveals that skin friction at the cylinder's surface can be maximized by increasing the particles concentration. For ternary nanoliquid, these are very sharp than hybrid and nano fluids. Further, the maximum values are observed when the concentration $\varphi_{Al_{2}O_{3},p1}=\varphi_{1}$ vary from 1% to 7% by holding $\varphi_{CuO,p2}=\varphi_{2}=3\%$ and $\varphi_{Cu,p3}=\varphi_{3}=3\%$. The skin friction values are minimized at saddle location while successive increment is obvious towards the saddle stagnation points. Similarly, Table 3 organized for the $\sqrt{R_{ex}}\;C_{Fy}$ with the same concentration ranges. Again, dominance in the skin friction with increasing concentration is noted and for saddle stagnation case these are minimum. Thus, saddle points are better to minimize the $\sqrt{R_{ex}}\;C_{Fx}$ and $\sqrt{R_{ex}}\;C_{Fy}$ wheraes; nodal points help to maximize $\sqrt{R_{ex}}\;C_{Fx}$ and $\sqrt{R_{ex}}\;C_{Fy}$.

**Table 2:** Skin friction computation $\sqrt{R_{ex}}\;C_{Fx}$ for varying parametric ranges.

Physical Parameters various Stages			Nodal Stagnation Region ${0.0<s}_{1}<1.0$			Saddle Stagnation Region ${-1.0<s}_{1}<0.0$		
			$\sqrt{R_{ex}}\;C_{Fx}$			$\sqrt{R_{ex}}\;C_{Fx}$		
			Nano	Hybrid	Ternary	Nano	Hybrid	Ternary
$\varphi_{1}$	$\varphi_{2}$	$\varphi_{3}$						
1%	3%	3%	1.295	1.384	1.412	1.167	1.260	1.290
3%			1.299	1.379	1.404	1.170	1.255	1.281
5%			1.300	1.373	1.393	1.171	1.248	1.270
7%			1.298	1.364	1.381	1.169	1.239	1.257
1%	2%			1.357	1.391		1.232	1.268
	4%			1.408	1.431		1.285	1.310
	6%			1.450	1.464		1.329	1.344
	8%			1.484	1.490		1.365	1.371
	1%	2%			1.356			1.231
		4%			1.379			1.255
		6%			1.397			1.274
		8%			1.410			1.288
**Computational Cost for the above Computation for Skin Friction**								
**Physical Parameters various Stages**			Nodal Stagnation Region ${0.0<s}_{1}<1.0$			Saddle Stagnation Region ${-1.0<s}_{1}<0.0$		
			$\sqrt{R_{ex}}\;C_{Fx}$			$\sqrt{R_{ex}}\;C_{Fx}$		
			Nano	Hybrid	Ternary	Nano	Hybrid	Ternary
$\varphi_{1}$	$\varphi_{2}$	$\varphi_{3}$						
1%	3%	3%	0.09s	0.08s	0.08s	0.14s	0.17s	0.14s
3%			0.13s	0.09s	0.20s	0.14s	0.13s	0.08s
5%			0.08s	0.11s	0.11s	0.11s	0.13s	0.11s
7%			0.08s	0.09s	0.08s	0.13s	0.13s	0.17s
1%	2%			0.11s	0.09s		0.09s	0.11s
	4%			0.08s	0.08s		0.19s	0.13s
	6%			0.13s	0.09s		0.13s	0.13s
	8%			0.08s	0.09s		0.09s	0.14s
	1%	2%			0.13s			0.11s
		4%			0.08s			0.14s
		6%			0.06s			0.09s
		8%			0.08s			0.13s

**Table 3:** Skin friction computation $\sqrt{R_{ex}}\;C_{Fy}$ for varying parametric ranges.

Physical Parameters various Stages			Nodal Stagnation Region ${0.0<s}_{1}<1.0$			Saddle Stagnation Region ${-1.0<s}_{1}<0.0$		
			$\sqrt{R_{ex}}\;C_{Fy}$			$\sqrt{R_{ex}}\;C_{Fy}$		
			Nano	Hybrid	Ternary	Nano	Hybrid	Ternary
$\varphi_{1}$	$\varphi_{2}$	$\varphi_{3}$						
0.01	0.03	0.03	1.197	1.227	1.253	0.705	0.597	0.506
0.03			1.200	1.227	1.269	0.699	0.601	0.520
0.05			1.201	1.226	1.273	0.697	0.806	0.537
0.07			1.200	1.222	1.268	0.700	0.619	0.557
0.01	0.02			1.254	1.276		0.597	0.540
	0.04			1.301	1.328		0.513	0.477
	0.06			1.340	1.358		0.448	0.427
	0.08			1.371	1.372		0.396	0.388
	0.01	0.02			1.253			0.598
		0.04			1.275			0.560
		0.06			1.291			0.531
		0.08			1.303			0.509
**Computational Cost for the above Computation for Skin Friction**								
**Physical Parameters various Stages**			Nodal Stagnation Region ${0.0<s}_{1}<1.0$			Saddle Stagnation Region ${-1.0<s}_{1}<0.0$		
			$\sqrt{R_{ex}}\;C_{Fy}$			$\sqrt{R_{ex}}\;C_{Fy}$		
			Nano	Hybrid	Ternary	Nano	Hybrid	Ternary
$\varphi_{1}$	$\varphi_{2}$	$\varphi_{3}$						
0.01	0.03	0.03	0.16s	0.38s	0.09s	0.11s	0.08s	0.11s
0.03			0.08s	0.08s	0.09s	0.09s	0.09s	0.11s
0.05			0.09s	0.06s	0.09s	0.09s	0.09s	0.11s
0.07			0.09s	0.08s	0.06s	0.08s	0.14s	0.11s
0.01	0.02			0.08s	0.09s		0.09s	0.11s
	0.04			0.08s	0.08s		0.09s	0.11s
	0.06			0.08s	0.09s		0.09s	0.14s
	0.08			0.08s	0.09s		0.11s	0.11s
	0.01	0.02			0.09s			0.09s
		0.04			0.09s			0.09s
		0.06			0.08s			0.11s
		0.08			0.11s			0.14s

The numerical computation for Nusselt number and corresponding computational cost is given in Table 4. The values of *Nu* at various parametric stages shown that ternary nanofluid has maximized heat transfer followed by simple mono nano and hybrid containing two types of nanoparticles. the results computed for $\varphi_{1},\varphi_{2},\varphi_{3},B_{i}$ and $R_{d}$. This indicates that ternary nanofluids are good for enhanced transfer which ultimately increase their industrial applications.

**Table 4:** Computation for Nusselt number with various parametric stages.

Physical Parameters various Stages					Nodal Stagnation Region ${0.0<s}_{1}<1.0$			Saddle Stagnation Region ${-1.0<s}_{1}<0.0$		
					|Nusselt Number|			|Nusselt Number|		
					Nano	Hybrid	Ternary	Nano	Hybrid	Ternary
$\varphi_{1}$	$\varphi_{2}$	$\varphi_{3}$	$B_{i}$	$R_{d}$						
1%	3%	3%	0.3	0.3	0.635	0.636	0.640	0.667	0.668	0.671
3%					0.640	0.641	0.650	0.671	0.672	0.678
5%					0.645	0.646	0.658	0.675	0.676	0.685
7%					0.649	0.651	0.666	0.678	0.679	0.691
1%	2%					0.638	0.642		0.669	0.672
	4%					0.641	0.645		0.671	0.674
	6%					0.644	0.648		0.674	0.677
	8%					0.648	0.652		0.676	0.680
	1%	2%								0.674
		4%								0.677
		6%								0.680
		8%								0.683
		3%	0.2		0.628	0.629	0.641	0.661	0.662	0.671
			0.4		0.641	0.642	0.653	0.672	0.673	0.680
			0.6		0.651	0.653	0.662	0.680	0.681	0.688
			0.8		0.660	0.661	0.670	0.686	0.687	0.694
			0.3	0.2	0.308	0.308	0.311	0.315	0.316	0.317
				0.4	1.350	1.356	1.405	1.503	1.508	1.549
				0.6	10.77	10.38	8.191	1.650	1.685	1.701
				0.8	1.962	1.949	1.856	1.710	1.703	1.853
**Computational Cost for the above Computation for Nusselt number**										
**Physical Parameters various Stages**					Nodal Stagnation Region ${0.0<s}_{1}<1.0$			Saddle Stagnation Region ${-1.0<s}_{1}<0.0$		
					Nusselt Number			Nusselt Number		
					Nano	Hybrid	Ternary	Nano	Hybrid	Ternary
$\varphi_{1}$	$\varphi_{2}$	$\varphi_{3}$	$B_{i}$	$R_{d}$						
1%	3%	3%	0.3	0.3	0.08s	0.19s	0.08s	0.06s	0.11s	0.13s
3%					0.08s	0.08s	0.05s	0.06s	0.08s	0.06s
5%					0.11s	0.06s	0.05s	0.05s	0.05s	0.05s
7%					0.11s	0.06s	0.09s	0.09s	0.08s	0.08s
1%	2%					0.05s	0.06s		0.08s	0.09s
	4%					0.08s	0.06s		0.06s	0.06s
	6%					0.06s	0.09		0.05s	0.08s
	8%					0.06s	0.03		0.05s	0.06s
	1%	2%								0.11s
		4%								0.17s
		6%								0.06s
		8%								0.06s
		3%	0.2		0.16s	0.05s	0.13s	0.11s	0.06s	0.16s
			0.4		0.06s	0.05s	0.05s	0.08s	0.06s	0.06s
			0.6		0.08s	0.08s	0.08s	0.05s	0.06s	0.05s
			0.8		0.05s	0.06s	0.08s	0.09s	0.05s	0.06s
			0.3	0.2	0.13s	0.08s	0.05s	0.08s	0.17s	0.06s
				0.4	0.13s	0.08s	0.08s	0.05s	0.20s	0.06s
				0.6	0.08s	0.06s	0.11s	0.13s	0.06s	0.08s
				0.8	0.06s	0.06s	0.06s	0.09s	0.06s	0.08s

### 4.4 Model and code validation

The current ternary nanoliquid model is compatible to those of the model discussed by Gangadhar et al. [37] by taking $\varphi_{1}=\varphi_{2}=\varphi_{3}=0.0\%$. The computation for the model is then performed and given in Table 5. It is obvious that the present model results using the developed code are perfectly aligned with the published data. This gives the reliability of the present work.

**Table 5:** The model and code validation with published data.

Quantities	Gangadhar et al. [37]		Present Results	
	$c_{1}$		$c_{1}$	
	−1/2	1/2	−1/2	1/2
$C_{fx}$	1.2302	1.2669	1.2303	1.26670
$C_{fy}$	0.0558	0.4991	0.557	0.4992

## 5 Conclusions

The influences of physical constraints (thermal radiations, nanoparticles strength, convective heat condition and saddle/nodal points) on the heat performance of ternary nanofluid are presented. The formulated problem dealt using numerical scheme and simulated the results. It is examined that.

• The motion of ternary nanofluid over the saddle and nodal areas drops by increasing the strength of Al_2_O_3_ nanoparticles form 1%–7% keeping fixed concentration of CuO and Cu as 4% and 6%, respectively.
• The ternary nanoliquid attained maximum velocity at nodal point against $s_{1}=0.2,0.4,0.6,0.8$ and it declines on saddle locations when $s_{1}$ changes from −0.2,−0.4,−0.6,−0.8.
• The temperature of ternary nanofluid boosted abruptly due to higher values of Biot number $B_{i}=0.1,0.2,0.3,0.4$ and it is optimum near the surface.
• The various concentration ranges of the nanoparticles positively affect the temperature on both saddle as well as nodal points.
• The shear drag at the surface can be maximized by increasing the nanoparticles strength and is optimum in the case of ternary nanofluid than nano and hybrid nanofluids.
• The values rate of heat transfer in ternary nanofluid in much higher than that of nano and hybrid nanoliquids due to strong thermal conductivity.

## Author contribution statement

Kamel SMIDA: Performed the experiments; Analyzed and interpreted the data; Wrote the paper. Abbasi Adnan: Conceived and designed the experiments; Analyzed and interpreted the data; Contributed reagents, materials, analysis tools or data. Muhammad Umer Sohail: Conceived and designed the experiments; Contributed reagents, materials, analysis tools or data. Iskander Tlili: Performed the experiments; Analyzed and interpreted the data; Wrote the paper. Asma Javed: Analyzed and interpreted the data; Contributed reagents, materials, analysis tools or data.

## Data availability statement

Data will be made available on request.

## Declaration of competing interest

There is no financial/competing interest regarding the publication of this work.
